# Cyber behaviour and personality nexus: clustering around security attitudes, FoMO, problematic social media use, and cognitive and personality traits?

**DOI:** 10.1098/rsos.251879

**Published:** 2026-09-09

**Authors:** Tourjana Islam Supti, Ala Yankouskaya, Mahmoud Barhmagi, Khaled M. Khan, Aiman Erbad, Raian Ali

**Affiliations:** ^1^ College of Engineering, Qatar University, Doha, Qatar; ^2^ School of Psychology, Bournemouth University, Poole, UK; ^3^ College of Science and Engineering, Hamad Bin Khalifa University, Doha, Qatar

**Keywords:** conscientiousness, neuroticism, need for cognition, problematic social media use, fear of missing out, cybersecurity, security attitude, clustering, persona

## Abstract

Our research examines whether cyber behaviours can be classified through personality factors. The study involved 642 participants, including 314 from the United Kingdom (UK) and 328 from the Arab Gulf Cooperation Council (GCC). Using conscientiousness, neuroticism and need for cognition (NFC) along with problematic social media use (PSMU), fear of missing out (FoMO) and security attitude (SA), we identified three clusters in each cultural context, which showed broad similarities. To enhance interpretation, clusters were translated into behavioural personas. The UK personas were ‘methodical achievers’, ‘reactive explorers’ and ‘engaged seekers’, while the GCC personas were ‘analytical protectors’, ‘reactive explorers’ and ‘hyper-connected defenders’. Developing these profiles and presenting them as visual personas was a central aim of the research. The clusters revealed consistent relationships between cyber behaviours and personal factors. High NFC and conscientiousness were linked to stronger SAs and lower PSMU and FoMO, whereas higher neuroticism predicted weaker SAs and elevated PSMU and FoMO. These findings underscore the utility of clustering approaches that integrate multiple behaviours rather than isolating one. By connecting personality with cyber tendencies, this study provides a foundation for personalized interventions, promoting cyber safety in a more comprehensive and culturally sensitive manner.

## 1. Introduction

The digital age has transformed how individuals interact, communicate and access information, creating new opportunities but also significant cybersecurity risks for both individuals and organizations [1]. As society becomes increasingly dependent on the Internet, cybercrime remains a widespread and evolving threat, with its full scale and economic impact difficult to measure yet widely acknowledged by experts [2]. While technological vulnerabilities are often emphasized, human behaviour remains a critical factor in security breaches, as individuals are subject to cognitive biases that can increase their susceptibility to cyber threats [3,4]. As people engage in various cyber behaviours, including social media use, cybersecurity practices and risk-taking activities, their online interactions and vulnerability to cyber threats are influenced by these behaviours. Problematic social media use (PSMU), fear of missing out (FoMO) and security attitudes (SAs) are key behaviours that shape how individuals engage with digital platforms and manage cyber risks. Social engineering tactics, such as phishing and pretexting, exploit psychological factors like trust, urgency and fear, impairing rational decision-making and making individuals prime targets for manipulation [5]. Research also shows that users often rely on social cues such as ratings and comments when downloading apps, sometimes ignoring antivirus alerts, which highlights how trust heuristics can override rational decision-making [6]. Understanding these psychological and behavioural factors is essential for developing effective interventions that mitigate cyber risks and promote safer, more responsible online behaviour.

Among the factors affecting cybersecurity behaviour, excessive or PSMU, commonly known as ‘social media addiction’ or ‘social media disorder’ [7,8], has gained significant attention. However, this terminology has faced criticism [9]. PSMU refers to the excessive use of social media that leads to adverse effects on different areas of an individual's daily life [10]. Additionally, the psychological phenomenon of FoMO has also been recognized for its influence on online behaviour and risk perceptions. FoMO is a psychological condition marked by a constant anxiety that others are experiencing enjoyable or fulfilling events that one is not part of [11,12]. These behaviours can create heightened emotional vulnerability, leading individuals to prioritize immediate gratification over long-term safety. Social media intensifies this vulnerability by acting as a space of continuous identity construction, where individuals seek social validation and belonging, increasing emotional dependency and susceptibility to risky online behaviours [13]. This emotional vulnerability is further reinforced by visual self-presentation practices on social media, where selfies function as a ‘pseudo-mirror’ that emphasizes visibility and social approval over self-reflection, increasing validation-seeking behaviour and weakening internal self-regulation [14].

A growing body of research highlights the significant role of PSMU and FoMO in shaping online behaviour and influencing cybersecurity-related attitudes and behaviours. A study by Sindermann *et al.* shows that certain design elements of social media platforms encourage users to spend more time on these platforms, which is strongly linked to PSMU [15]. PSMU is linked to factors such as depression, FoMO, the need to belong and increased social media use [16]. A study by Weaver & Swank [17] reveals that higher levels of FoMO and PSMU are associated with lower self-esteem, mindfulness and life satisfaction among undergraduates. Çetinkaya *et al.* identify the fear of missing activity as a key driver of PSMU and intrusion, while the fear of missing experience shows weaker effects [18]. These findings further demonstrate a bidirectional relationship, whereby PSMU also intensifies FoMO, creating a self-perpetuating cycle of compulsive engagement. During the pandemic, FoMO shifted from physical events to online activities, continuing to negatively impact well-being, including sleep deprivation and reduced focus [19]. Furthermore, FoMO has been shown to increase stress levels among social media users [20–22]. PSMU is also strongly correlated with cybercrime victimization, with increased levels raising the risk of becoming a victim [23]. The widespread sharing of information on social networks enhances the chances of cyberattacks by hackers [24]. In parallel, studies on official microblogs demonstrate that user engagement, measured through likes, comments and shares, significantly contributes to influence and information diffusion, underscoring the centrality of interaction patterns in shaping online behaviour [25]. A study by Deutrom *et al.* found that problematic Internet use is associated with poorer cybersecurity behaviours [26]. Additionally, employees with higher FoMO levels tend to have lower information security awareness (ISA), demonstrating poorer knowledge, more negative attitudes and riskier behaviours [27]. A cyclical relationship between FoMO-centric design and privacy-compromising behaviour has also been observed, where individuals, despite expressing privacy concerns, engage in risky behaviours owing to the pressure to participate [28]. Furthermore, an individual's SA, belief in the importance of cybersecurity and intention to adopt secure practices significantly influence protective behaviours. Those with a stronger belief in cybersecurity are likely to adopt protective behaviours despite the emotional pull of PSMU and FoMO. However, it is important to note that social media use does not always result in negative outcomes. A study examining Facebook adoption across 72 countries from 2008 to 2019 found a slight association with improved psychological well-being, particularly among younger users, with no evidence of widespread harm [29]. This suggests that the effects of social media on well-being and behaviour may be nuanced, depending on usage patterns, platform features and individual differences.

Individual differences, especially personality traits, play a key role in social media engagement, digital boundary management and perceptions of security, yet their relationship with digital security remains largely underexplored. Personality traits are enduring characteristics that influence an individual's thinking, feeling and behaviour patterns. These traits are often described through frameworks like the Big Five Personality Traits (openness, conscientiousness, extraversion, agreeableness and neuroticism) [30,31]. Research has shown that certain personality traits are significantly associated with cybersecurity behaviours. Attitudes towards security recommendations are a multi-dimensional construct, with employees evaluating security policies based on their perceived legitimacy, effectiveness and rigour [32]. For instance, conscientiousness, agreeableness and openness are key traits influencing how individuals engage with digital security, with conscientiousness being the strongest predictor of secure behaviour [33]. Neuroticism, on the other hand, has been linked to increased susceptibility to phishing attacks. At the same time, openness to experience is associated with more relaxed privacy settings and a greater tendency to share personal information, making individuals more vulnerable to privacy breaches [34]. Furthermore, conscientiousness, agreeableness, emotional stability and risk-taking propensity influence ISA [35]. Specifically, conscientious individuals, who tend to be hardworking and detail-oriented, are more likely to engage in secure online practices [36]. Conscientiousness is strongly associated with secure online behaviours, as individuals high in this trait are likelier to follow cybersecurity practices diligently.

In the context of social media use, neuroticism and impulsivity are indirectly associated with PSMU through FoMO and the increased use of social media driven by design features [15]. People with high levels of neuroticism often report feelings of excessive or addictive social media use, suggesting a greater perceived dependency on social media [37]. Neuroticism is associated with higher PSMU severity, particularly among emotionally vulnerable individuals [38]. A study by Alshakhsi *et al.* found that neuroticism is significantly associated with PSMU, with FoMO mediating this relationship fully in the United Kingdom (UK) and partially in the Arab region's sample [39]. Additionally, neuroticism has been linked to FoMO, with studies revealing that it significantly increases susceptibility to social influence [40]. While neuroticism is strongly positively associated with FoMO, conscientiousness shows a small negative association with FoMO across various analyses [41]. This suggests that individuals with higher neuroticism are more likely to experience FoMO, potentially leading to increased social media use and reduced engagement with secure online practices. Personality traits, such as neuroticism and conscientiousness, significantly influence how individuals perceive and respond to both social media use and digital security practices. These findings highlight the need to consider individual differences when developing interventions aimed at promoting secure behaviour and managing social media dependency.

In addition to personality traits, the need for cognition (NFC) also plays a significant role in decision-making processes related to cybersecurity. NFC refers to an individual's tendency to engage in and enjoy effortful cognitive activities, such as thinking critically and solving complex problems [42]. Likewise, cognitive styles, including NFC, need for affect and faith in intuition (FII), also play a role in decision-making processes related to cybersecurity [43,44]. People with high NFC, for example, are more likely to seek out detailed information, making them more cautious in decision-making [42], which can lead to adopting more secure measures. On the other hand, individuals with high reliance on intuition may make more impulsive decisions, relying on heuristics that overlook updated information [45], potentially increasing their online vulnerability. The NFC significantly influences decision-making in cybersecurity, as individuals high in NFC are more inclined to seek out detailed information and think critically before making decisions. This thoughtful approach leads to more secure online behaviours, as they are less likely to engage in risky actions without understanding the potential consequences. Additionally, individuals with high NFC are less susceptible to emotional impulses, like FoMO, and are better at managing PSMU. By prioritizing rational analysis over impulsivity, those with high NFC can reduce the impact of social media pressures and enhance their cybersecurity attitudes, making more informed and secure choices.

While previous studies have provided valuable insights, they often examine these dimensions in isolation, overlooking their potential interplay. According to recent systematic literature reviews on cybersecurity and human factors [46], as well as research on PSMU [47], the majority of studies in this area have focused on Western contexts, which is acronymized as WEIRD (Western, educated, industrialized, rich and democratic) [48], with a limited exploration of cybersecurity attitudes and cyber behaviours within the Middle Eastern, particularly Arab, populations. Cultural norms, social influences and varying levels of cybersecurity awareness can shape how individuals perceive vulnerability and engage in cyber behaviours. Research suggests that sociocultural factors, such as individualism–collectivism and tightness–looseness, influence risk perception and the use of base rate information in assessing cyber threats [49]. Individuals in cultures with high uncertainty avoidance may be more cautious and vigilant in adopting secure online practices than those in low uncertainty avoidance cultures [50].

By applying clustering techniques, this research aims to identify distinct cyber behaviour profiles based on personality traits (conscientiousness and neuroticism), cognitive style (NFC) and behavioural factors (PSMU and FoMO) in the UK and Arab regions. The study examines whether different cyber behaviours can be grouped similarly across cultural contexts based on shared personal characteristics. Understanding these clusters provides deeper insights into the interplay of psychological and behavioural factors in cybersecurity, facilitating the development of more culturally sensitive interventions. Additionally, this research fills a gap in the literature by integrating cross-cultural differences into cybersecurity behaviour analysis, particularly in non-WEIRD population samples.

The primary aim of this study is to identify distinct profiles of social media users based on personality traits (conscientiousness and neuroticism), cognitive style and cyber behaviour factors. Conscientiousness and neuroticism were selected as focal personality dimensions owing to their strong theoretical relevance to self-regulatory behaviour, emotional reactivity and susceptibility to problematic digital engagement, which are central to social media disorder and cyber behaviour outcomes [51]. Conscientiousness is typically associated with self-discipline, responsibility and effective self-regulation [52], which may facilitate more controlled and adaptive engagement with social media. By contrast, neuroticism reflects emotional instability and heightened sensitivity to stress [53], which may increase vulnerability to FoMO and problematic patterns of social media use.

Based on this foundation, we can formulate the following research question, on both Arab and British samples:

**RQ1:** *Can we identify distinct cyber behaviour profiles among individuals based on their personality traits (conscientiousness and neuroticism), cognitive style (NFC) on the one hand and their cyber behavioural factors (PSMU, FoMO and SA) on the other?*

## 2. Research method

Our study is part of a more extensive investigation examining the factors that shape cybersecurity attitudes and cyber behaviours. Both the Arabic and English versions of these scales and questions are available on the Open Science Framework (OSF), with the link provided in the ‘Data accessibility’ section of this manuscript.

### 2.1. Participants and procedure

Participants for this study were recruited from the Gulf Cooperation Council (GCC) region and the UK through TGM Research (https://tgmresearch.com/), a company specializing in research data collection. These two cultural contexts were selected for their contrasting societal values and moral principles, offering a rich basis for comparative analysis [54]. The survey was designed and administered via SurveyMonkey (www.surveymonkey.com), a platform for creating and distributing questionnaires. To ensure that the survey questions were clear and accurate, the research team followed an iterative development process. The survey was first drafted in English and then translated into Arabic by two team members using the recommended back-translation method [55]. A pilot test was then conducted with a small group of participants to identify and address any ambiguities or unclear wording.

Eligibility criteria required participants to be over 18 years old and born and currently residing in either the UK (England, Scotland, Wales and Northern Ireland) or one of the Arab GCC countries (Saudi Arabia, Qatar, Bahrain, Kuwait, Oman and the UAE). Additionally, participants from the GCC region had to explicitly self-identify as Arabs in terms of cultural norms and values. Informed consent was obtained from all participants, who could withdraw from the survey at any time. To ensure data quality, attention checks were embedded within the survey, and participants who failed to pass the attention checks and completed the survey too quickly or provided monotonous answers were excluded. Those who successfully completed the survey were compensated for their time.

Ethical approval for the study was granted by the Institutional Review Board at the institution of the last author. Participants identifying as non-binary were excluded from the analysis owing to their small sample size in both the Arab and UK datasets. Similarly, participants aged 60 and above were excluded from the UK sample to address the uneven age distribution across the two samples, as we could not get any participants in that age group from the Arab sample. While older individuals participated in the UK sample, only 7% of the population in many Arab countries falls within this age group [56]. Limiting the age range to 18–60 years ensured consistency. The final dataset included 642 participants, comprising 314 from the UK and 328 from the Arab GCC region.

### 2.2. Measurements

#### 2.2.1. Demographic measure

Participants were asked to provide their age and gender. Age was recorded as a continuous variable in years, while gender was collected through an open-text field and then coded accordingly.

#### 2.2.2. Big Five Inventory

The study employed the Big Five Inventory (BFI-10) to assess personality traits [57]. This abbreviated version of the Big Five Inventory evaluates five dimensions of personality: extraversion indicates the intensity of being outgoing and interactive; introversion, which is the opposite of extraversion; agreeableness identifies how friendly and trusting the person is. Neuroticism indicates the stability of emotion; openness measures how open to experience the user is; conscientiousness indicates that the person is focused on goals and determined, with two items dedicated to each trait. Participants rated their agreement with each statement on a five-point Likert scale, ranging from ‘1 = strongly disagree’ to ‘5 = strongly agree’. Some items are reverse-scored to control for response bias. The final score for each personality trait is calculated by summing the responses across all relevant items. In this study, we used only two personality traits: conscientiousness and neuroticism. The theoretical range for conscientiousness and neuroticism in the UK sample was 2–10, whereas in the Arab sample, the range was 3–10 for conscientiousness and 2–10 for neuroticism.

#### 2.2.3. Need for cognition

NFC was measured using the five-item subscale of the Rational Experiential Inventory (REI-10), which also includes a subscale for FII [58]. This scale is a shortened version of the original developed by Cacioppo & Petty [42], and participants rated their agreement with statements on a five-point Likert scale ranging from ‘1 = completely false’ to ‘5 = completely true’. The theoretical range was 6–25. An example item from the NFC scale is, ‘*I don't like to have to do a lot of thinking.*’ Several items were slightly adjusted for clarity and to ensure consistency between the English and Arabic versions of the scale. For example, the original NFC item ‘*Thinking hard and for a long time about something gives me little satisfaction*’ was modified to ‘*Thinking hard and for a long time about something gives me some satisfaction*’ to enhance its understanding of the study's cultural and linguistic context. The NFC total score is calculated by summing the responses to all scale items after reverse-scoring the negatively worded items, with higher scores indicating a greater preference for effortful thinking. The scale showed very good internal reliability across both samples, with Cronbach's alpha of 0.82 in the UK sample and 0.70 in the Arab sample.

#### 2.2.4. Problematic social media use

PSMU was measured using the original English version and a translated Arabic version of the ‘Social Media Disorder Scale’ [59]. The scale consists of nine items, one of which is ‘*Have you ever found yourself unable to think of anything else but the moment when you'll be able to use social media again?*’ Participants responded to each item on a five-point Likert scale, ranging from ‘1 = never’ to ‘5 = always’. The theoretical range for total PSMU was from 9 to 40. The total PSMU score was calculated by summing participants’ responses, with higher scores indicating greater levels of social media disorder. In previous studies, the scale has shown very good internal consistency, with Cronbach's alpha ranging from *α* = 0.76 to 0.82. In our study, Cronbach's alpha was 0.87 for the UK sample and 0.84 for the Arab sample, indicating very good reliability.

#### 2.2.5. Fear of missing out

To measure the concept of FoMO, participants were provided a definition: ‘FoMO, the fear of missing out, refers to the fear of not being able to know what is happening (whether on social media or in real-world) and participate in it and taking opportunities.’ Participants were then asked to rate their agreement with the statement: ‘*I experience FoMO regarding what is happening on social media.*’ Responses were collected using a 10-point Likert scale, where ‘1 = strongly disagree’ and ‘10 = strongly agree’, with higher scores indicating a stronger FoMO.

This approach was adopted in line with the analytical objectives of the study. Riordan *et al.* showed that a single-item FoMO measure performs comparably to a 10-item scale on key psychometric criteria, including concurrent validity and reliability [60]. In the context of clustering, where the goal shifts from precise measurement of a latent construct to the relative positioning of participants, this level of validity is sufficient. Accordingly, the trade-off between measurement granularity and methodological rigour in cluster composition was resolved in favour of the latter.

Furthermore, all variables included in the clustering procedure are represented as single aggregated scores (e.g. composite means or sums), meaning that each variable enters the cluster solution as one numeric value. In this context, the FoMO single-item measure is not a methodological compromise; it is functionally identical to the other variables in how it contributes to clustering. The distinction between a single- and a multi-item scale becomes less relevant at the point of analysis, as multi-item scales are themselves reduced to a single value before entering the clustering algorithm.

#### 2.2.6. Security attitude

The SAs (SA-6) scale, developed by Faklaris *et al*. [61], is a validated six-item instrument to assess individuals' attitudes towards cybersecurity. Extensive empirical research supports the scale's reliability and validity, revealing various responses. Participants rated their agreement with statements like ‘*I actively seek opportunities to learn about security measures that apply to me*’ and ‘*I am highly motivated to take all necessary steps to protect my online data and accounts*’ using a five-point Likert scale (1 = strongly disagree to 5 = strongly agree). The SA-6 scale has been shown to correlate significantly with both self-reported security intentions and actual secure behaviours, confirming its utility in measuring and comparing attitudes towards adopting recommended security practices. In the original validation study, the SA-6 scale demonstrated strong internal consistency with a Cronbach's alpha of 0.84 [61]. In our study, the scale yielded a Cronbach's alpha of 0.87 for the UK participants and 0.79 for the Arab participants, reflecting very good internal consistency. The total SA score for each participant was calculated by summing the individual item scores, providing a standardized measure of SAs.

#### 2.2.7. Validity of cross-cultural comparisons

Measurement invariance across UK and Arab participants was tested separately for the NFC, SA and PSMU scales using multiple-group confirmatory factor analysis. Because the items were ordinal Likert-type indicators, models were estimated using the weighted least squares mean- and variance-adjusted estimator (WLSMV). Invariance was evaluated through a sequence of nested models with progressively stronger equality constraints across groups [62]. Decisions about invariance were based primarily on changes in approximate fit indices between nested models. This choice follows recommendations that ΔCFI, together with ΔRMSEA and ΔSRMR, provides a more stable basis for evaluating invariance [63]. Invariance was considered supported when the deterioration in fit across nested models remained within conventional cut-offs (ΔCFI ≤ 0.010, ΔRMSEA ≤ 0.015 and ΔSRMR ≤ 0.030 for loading-related comparisons, with a stricter ΔSRMR ≤ 0.010 often applied for intercept/threshold-related constraints) [64].

For NFC, the change from the configural model to the threshold invariance model was small (ΔCFI = –0.007, ΔRMSEA = –0.065, ΔSRMR = 0.001), supporting threshold invariance across the Arab and UK groups. The additional constraint of equal factor loadings produced negligible further change in model fit (ΔCFI = –0.001, ΔRMSEA = –0.010, ΔSRMR = 0.0002), supporting invariance of factor loadings given equal thresholds. Imposing equality constraints on residual variances resulted in ΔCFI = –0.010, ΔRMSEA = 0.004 and ΔSRMR = 0.004, indicating that this more restrictive model was also acceptable. Overall, these supported the measurement invariance of the NFC scale across the two cultural groups.

For the PSMU, the comparison between the configural and threshold models showed only trivial deterioration in fit (ΔCFI = –0.002, ΔRMSEA = –0.011, ΔSRMR = 0.001), supporting threshold equivalence across groups. Constraining factor loadings in addition to thresholds resulted in almost no further reduction in fit (ΔCFI = –0.002, ΔRMSEA = –0.0002, ΔSRMR = 0.002), indicating that the strength of the item–factor relations was comparable across the Arab and UK samples. When residual variances were additionally constrained, the observed changes remained modest (ΔCFI = –0.007, ΔRMSEA = 0.010, ΔSRMR = 0.005). Overall, the results are consistent with cross-group invariance for PSMU.

For the SA scale, the transition from the configural model to the threshold-constrained model was associated with only a trivial change in fit (ΔCFI = –0.002, ΔRMSEA = –0.004, ΔSRMR = 0.001), supporting the equality of thresholds across groups. Introducing equality constraints on the factor loadings resulted in a small additional reduction in fit (ΔCFI = –0.004, ΔRMSEA = 0.011, ΔSRMR = 0.003), which remained within conventional criteria and therefore supported invariance of factor loadings given equal thresholds. Constraining residual variances in the final step led to only modest further change (ΔCFI = –0.003, ΔRMSEA = 0.004, ΔSRMR = 0.008), again remaining within commonly accepted limits. These results supported the measurement invariance of the SA scale across the two cultural groups.

### 2.3. Data pre-processing

As an initial step in our data analysis, we examined the relationships among all the variables across both the UK and Arab samples. These correlations, which provided insights into the interplay between cyber behaviours and individual differences before clustering, are illustrated in figure S1 in the Appendix provided in the ‘Data accessibility’ section. Prior to conducting cluster analysis, we implemented a two-stage data pre-processing procedure. First, we standardized the datasets for both samples using the scale() function in R, which normalizes the numeric matrix by adjusting its columns. To determine whether the data were suitable for clustering, we assessed clustering tendency through visual analysis of two matrices. The first matrix represented correlation-based distances between data points in the original dataset, computed using the Spearman method via the get_dist() function in R. The second matrix, generated using randomly assigned values, maintained the same dimensions as the original dataset. A comparative visual inspection of these matrices confirmed that our data exhibited an appropriate structure for clustering, as presented in figure S2 in the Appendix provided in the ‘Data accessibility’ section.

Before clustering, variables were standardized (*z*-scores) to ensure comparability across different scales. Cluster solutions were evaluated using interpretability and cluster separation, and all cluster characteristics were described relative to the sample mean (i.e. higher or lower than average values).

Additionally, group differences were examined using Welch's *t*-tests, and Bayesian independent samples *t*-tests were conducted to estimate Bayes factors (BF_10_) using jamovi (v.2.7.24.0). Bayesian analyses were conducted using default prior settings (Cauchy prior, *r* = 0.707), and BF₁₀ values were interpreted according to established conventions (e.g. anecdotal, moderate, strong and very strong evidence). Correlation analyses were performed using JASP (v.0.96), with results reported in figure S1 in the Appendix provided in the ‘Data accessibility’ section. Descriptive interpretation of cluster characteristics was also conducted in JASP, with full details presented in table S1 in the Appendix (see OSF link in the ‘Data accessibility’ section).

### 2.4. Clustering approach

We applied partition clustering to group observations into distinct clusters based on their similarities within each sample. The variables used for clustering included conscientiousness, neuroticism, NFC, PSMU, FoMO and SA. For this purpose, we implemented *k*-means clustering, an unsupervised machine learning algorithm that divides data into a set number of clusters, denoted by *k*. This algorithm aims to maximize the similarity within each cluster and minimize the dissimilarity between different clusters. To carry out this analysis, we utilized the Hartigan–Wong method [65], which minimizes the within-cluster variance by calculating the sum of squared Euclidean distances between observations and the centroids of their respective clusters. Each observation is assigned to a cluster so as to minimize the squared distance to its assigned centroid, which was computed using the *k*-means() function in R's stats package.

As *k*-means clustering requires a predefined number of clusters, we determined the optimal number of clusters, *k*, using the NbClust package in R. This package offers 30 indices to suggest the most suitable clustering solution by evaluating various combinations of *k* values, distance metrics and clustering techniques [66]. To ensure reliable results, we undertook three additional steps. First, we tested different *k*-values to compare clustering outcomes and avoid arbitrary cluster selections. Second, in light of *k*-means’ sensitivity to the initial random placement of cluster centroids [67], we ran the clustering algorithm five times with different initial cluster centre assignments, choosing the configuration with the lowest within-cluster sum of squares. For stability, we used 15, 25, 35, 45 and 55 random initializations with 1000 iterations per run. Lastly, recognizing the potential impact of outliers on clustering performance, we carefully examined the data for outliers prior to performing the clustering.

We rerun the clustering procedure without FoMO. Cluster solutions with and without FoMO were compared using the adjusted rand index (ARI) to assess the stability of the clustering structure. (The detailed results of the cluster comparison are reported in the ‘Results’ section.)

## 3. Results

### 3.1. Dataset characteristics

The overview of the UK and Arab sample participants is presented in table 1 for this study. A Welch's *t*-test was conducted to examine the null hypothesis that the means of the two groups are equal. Additionally, evidence for the alternative hypothesis was assessed using the BF_10_ with default priors [69]. Arab participants tended to score slightly higher in conscientiousness, indicating a more organized and goal-oriented approach compared with UK participants. This difference in conscientiousness may reflect contrasting ways of managing tasks and responsibilities. UK participants, on the other hand, were more neurotic and had higher levels of NFC, suggesting they may be more emotionally reactive and cognitively engaged in problem-solving. While UK participants exhibited a heightened cognitive focus, Arab participants showed stronger connections to PSMU, FoMO and SA, highlighting differences in how each group approaches both emotional and cognitive aspects of their lives.

**Table 1.** Descriptive statistics analysis of all the variables for the UK and Arab samples.

variables		UK (*N* = 314)	Arab (*N* = 328)	*t*-test (^a^*W*, *p*-value, ^b^BF_10_)
gender				
	males	131 (42%)	185 (56%)	
	females	183 (58%)	143 (44%)	
age				
	males	38.99 (12.22)	37.88 (10.08)	–0.85, *p* = 0.397, BF_10_ = 0.18
	females	36.95 (12.08)	32.64 (9.31)	–3.64, *p* < 0.001, BF_10_ = 43.48
conscientiousness		7.59 (1.75)	7.99 (1.62)	3.03, *p* = 0.003, BF_10_ = 7.85
neuroticism		6.48 (2.09)	5.39 (2.02)	–6.76, *p* < 0.001, BF_10_ = 14.19
NFC		17.44 (3.98)	16.21 (3.75)	–4.02, *p* < 0.001, BF_10_ > 100
PSMU		16.83 (5.78)	22.75 (6.73)	11.98, *p* < 0.001, BF_10_ = 34.37
FoMO		4.01 (2.69)	5.40 (2.51)	6.79, *p* < 0.001, BF_10_ = 15.86
SA		21.18 (4.48)	24.47 (3.17)	10.70, *p* < 0.001, BF_10_ = 37.39

^a^Negative values for Welch's test indicate a smaller mean for the Arab sample than the UK sample.

^b^According to Lee & Wagenmakers [68] Classification scheme, BF_10_ > 100 provides extreme evidence for the alternative hypothesis (H1): 30–100 – very strong evidence; 10–30 – strong evidence; 3–10 moderate evidence; 1–3 and 1/3–1 – anecdotal evidence; 1 – no evidence.

### 3.2. Results of clustering analysis

**Figure 1.**
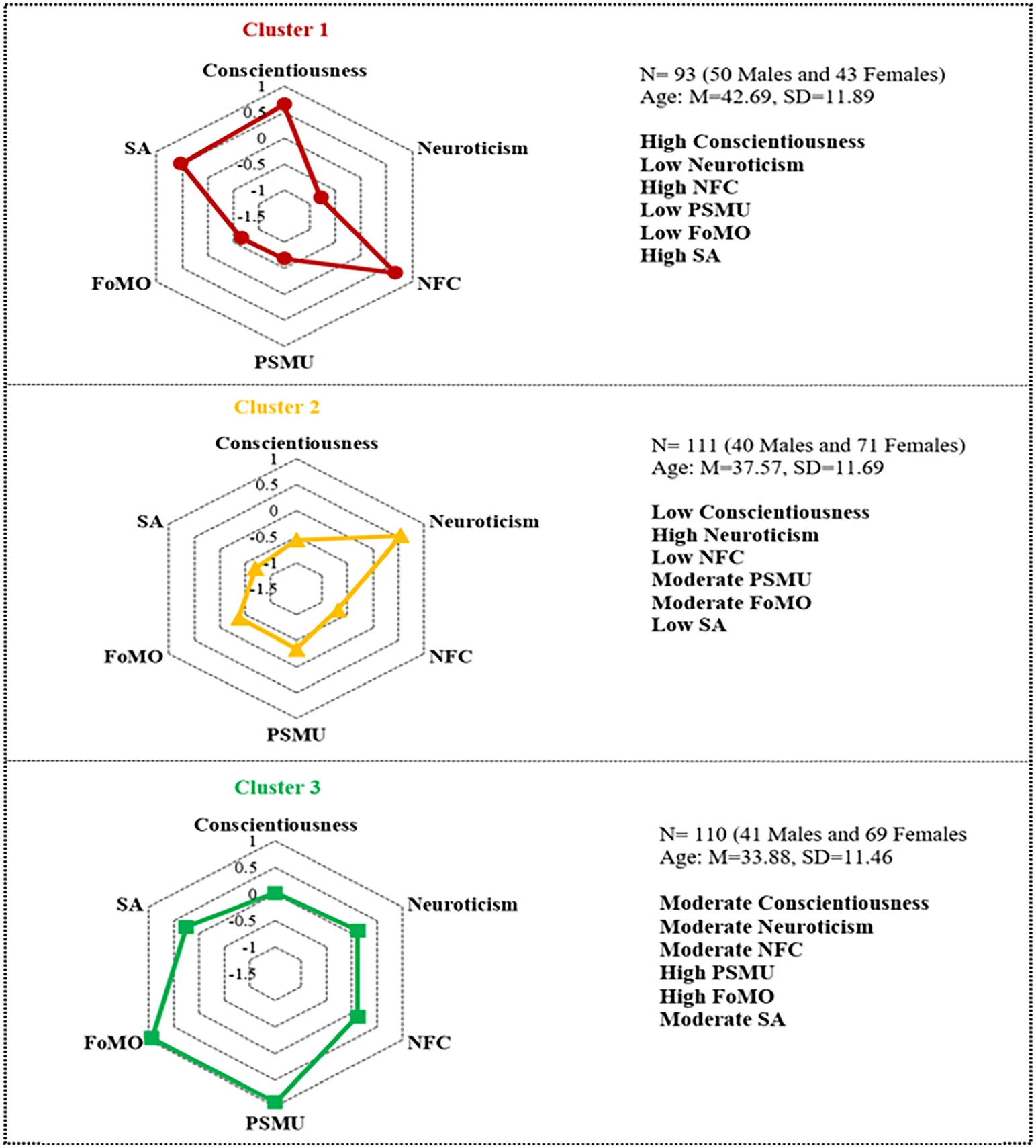
Cluster characteristics in the UK sample.

**Figure 2.**
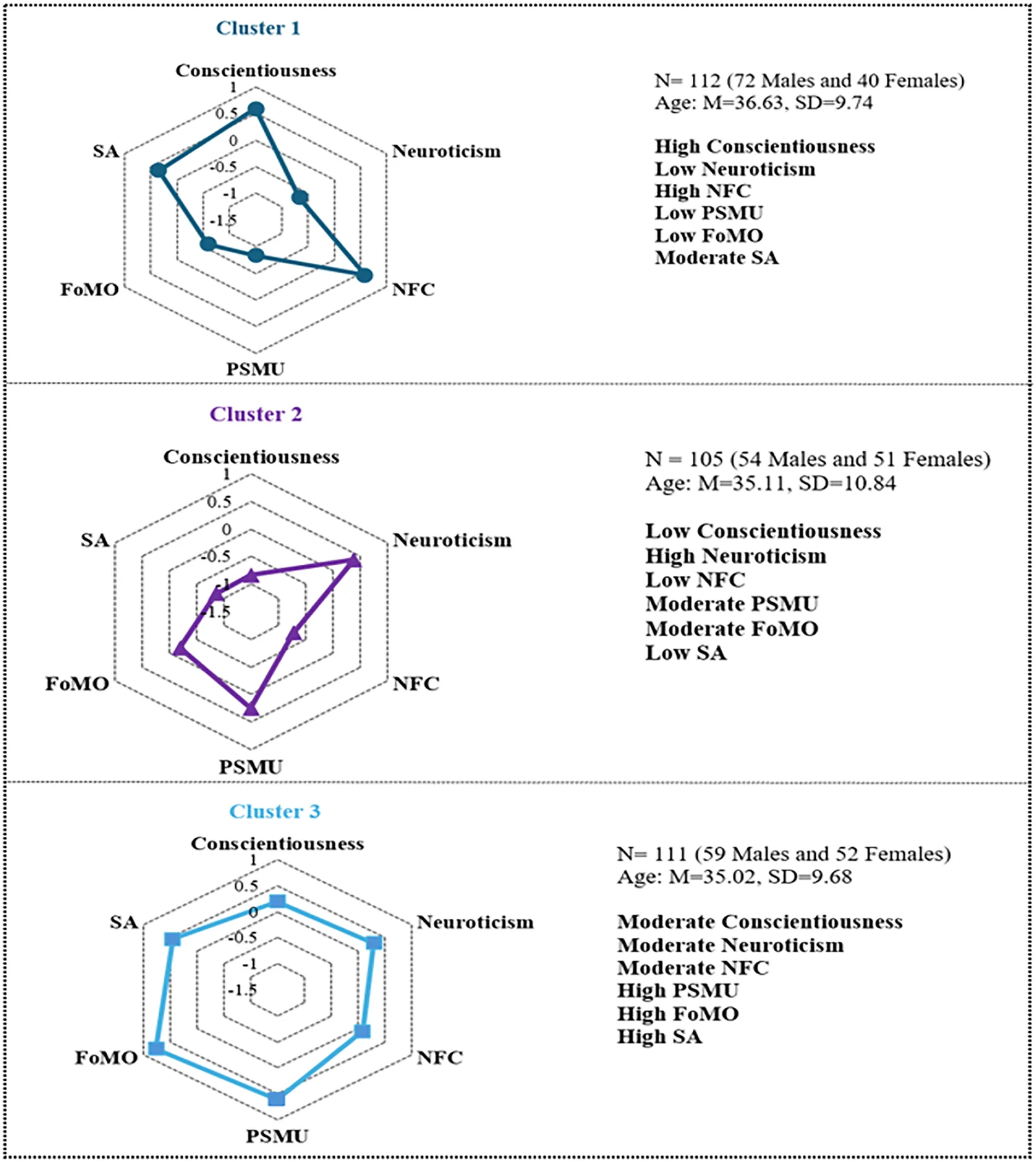
Cluster characteristics in the Arab sample.

Our analysis sought to determine the optimal number of clusters using multiple complementary approaches. The NbClust package, which evaluates 30 indices, indicated that *k* = 3 was the most frequently supported solution in both samples (UK: 13 indices; Arab: 9 indices), followed by *k* = 2 (UK: 5 indices; Arab: 6 indices). The optimal number of clusters was determined using visual inspection of the elbow, silhouette and gap statistic with 500 bootstrap samples. Across these methods, *k* = 3 emerged as the most supported solution. Overall, both theoretical and empirical evidence support retaining the 3-cluster solution, as it captures meaningful heterogeneity in how psychological traits, FoMO and PSMU interact. By contrast, a 2-cluster solution would oversimplify these patterns by forcing individuals into a binary structure and obscuring the intermediate profiles that are consistently observed across variables (see Sections C, D and E in the Appendix at the OSF link provided in the ‘Data accessibility’ section). Figures 1 and 2 present the defining characteristics of these clusters. For the UK sample, three distinct clusters were identified based on conscientiousness, neuroticism, NFC, PSMU, FoMO and SA. All cluster characteristics are described relative to standardized scores (*z*-scores), where positive values indicate above-average levels and negative values indicate below-average levels within the sample.

Cluster 1 (*N* = 93) is characterized by high levels of conscientiousness and NFC, coupled with low neuroticism, PSMU and FoMO. Members of this cluster display the strongest SAs, suggesting a high level of vigilance and engagement with secure behaviours. Cluster 2 (*N* = 111) exhibits low conscientiousness, high neuroticism and low NFC. Individuals in this cluster show moderate levels of PSMU and FoMO, indicating some dependence on social media but not at extreme levels. Their SA is the weakest among the three clusters, suggesting greater vulnerability to cybersecurity risks. Cluster 3 (*N* = 110) is defined by moderate levels of conscientiousness, neuroticism and NFC, alongside high levels of PSMU and FoMO. This cluster shows average SAs, balancing some secure behaviours with potential vulnerabilities owing to their high engagement with social media and increased anxiety about missing out.

Similarly, for the Arab sample, three distinct clusters were identified based on conscientiousness, neuroticism, NFC, PSMU, FoMO and SA. Cluster 1 (*N* = 112) is characterized by high conscientiousness and NFC, along with low neuroticism, PSMU and FoMO. Members of this cluster exhibit moderate SA, suggesting a reasonable level of engagement with secure behaviours. Cluster 2 (*N* = 105) displays low conscientiousness, high neuroticism and low NFC. Individuals in this cluster report moderate levels of PSMU and FoMO, with weak SA, indicating a greater vulnerability to cybersecurity risks. Cluster 3 (*N* = 111) features moderate conscientiousness, neuroticism and NFC, combined with high PSMU and FoMO. This cluster demonstrates strong SA, which may reflect their heightened engagement with cybersecurity practices despite significant social media dependency and FoMO.

The comparison of clustering solutions with and without FoMO revealed cross-cultural differences in how FoMO contributes to the latent structure of psychological profiles. In the UK sample, a moderate-to-high agreement was observed between the two solutions (ARI = 0.67), indicating that the clusters were largely recoverable without FoMO and not overly dependent on this variable (i.e. close to the suggested robustness threshold of ARI > 0.70). By contrast, the Arab sample showed lower agreement (ARI = 0.303), indicating that FoMO contributes substantially to cluster formation.

Importantly, we also examined the nature of these differences. In the UK sample, changes were primarily limited to the composition of the high-risk cluster, which shifted from a combined FoMO-behavioural vulnerability profile to a more behaviourally driven profile when FoMO was removed, while the overall structure remained stable. In the Arab sample, the removal of FoMO led to more substantial restructuring and split into distinct emotion- and behaviour-based clusters. These findings demonstrate that FoMO plays a refining role in the UK sample, but a more structurally influential role in the Arab sample. All details of the clustering results without FoMO can be found in Section F of the Appendix, available through the OSF link provided in the ‘Data accessibility’ section.

For the UK, one-way ANOVA results showed that FoMO (*F* = 143.34, *η*² = 0.480), PSMU (*F* = 142.70, *η*² = 0.479) and SA (*F* = 61.50, *η*² = 0.283) are significantly differentiated clusters (all *p* < 0.001). For Arab, one-way ANOVA results indicated that PSMU (*F* = 100.91, *η*² = 0.383), SA (*F* = 85.01, *η*² = 0.343) and FoMO (*F* = 81.27, *η*² = 0.333) all significantly contributed to cluster separation (all *p* < 0.001). No single variable dominated cluster differentiation, indicating a more balanced multi-dimensional structure in which FoMO functions as one of several comparable contributors.

#### 3.2.1. Clustering quality evaluation

To characterize the distinct profiles of each cluster, we examined the cluster centroids, representing the mean values of each variable within each cluster. All variables were *z*-standardized relative to the overall sample before clustering; therefore, the centroid values reflect standardized mean differences relative to the overall sample mean. Positive values indicate that a cluster scores above the sample average, whereas negative values indicate below-average scores. This approach provides a descriptive interpretation of cluster characteristics. The cluster characteristics are presented in table S1 in the Appendix and further visualized through interval plots (see the Appendix, table S1, figures S5 and S6), demonstrating the relative positioning of each cluster across the studied variables.

To assess clustering quality, we used the silhouette coefficient, which was 0.19 for the UK sample and 0.16 for the Arab sample. Although the silhouette scores are relatively low, this is not unusual in psychological and attitudinal clustering research. In these contexts, the constructs being measured tend to be continuous and overlapping rather than forming clearly separated groups. As a result, clustering methods often produce modest separation between profiles. For instance, Toffalini *et al.* showed that, in a large review of 191 studies, clustering solutions in psychology are frequently less stable and tend to reflect weakly differentiated groups, especially when sample sizes are moderate, and constructs are dimensional [70]. Further supporting this, simulation work by Toffalini *et al.* showed that commonly used clustering algorithms often struggle to clearly distinguish between one and two clusters under realistic psychological data conditions [71]. Taken together, these findings suggest that lower silhouette values should be expected in this type of data. Rather than indicating invalid clustering, they more likely reflect the inherent structure of psychological constructs, where boundaries between groups are naturally less distinct.

To evaluate the robustness of the retained 3-cluster solution, a model-based latent profile analysis (LPA) was conducted on the same standardized indicators in the Arab sample. The three-profile solution showed interpretable patterns: Profile 1 reflected lower conscientiousness, higher neuroticism, lower NFC, moderately higher PSMU and lower SA; Profile 2 showed higher conscientiousness and NFC, elevated PSMU and FoMO, and the highest SA; Profile 3 was characterized by relatively high conscientiousness, low neuroticism, higher NFC, and low PSMU and FoMO. Comparison between *k*-means and LPA solutions indicated moderate agreement (ARI = 0.47). Cross-classification showed that *k*-means Cluster 1 aligned primarily with LPA Profile 1, and Cluster 2 with Profile 3. By contrast, Cluster 3 was distributed across Profiles 1 and 2, indicating relatively lower stability; however, it remained theoretically meaningful, as it captured individuals with mixed characteristics. This is consistent with evidence suggesting that personality traits can combine in nonlinear ways to produce heterogeneous behavioural patterns [72,73].

The same 3-cluster solution was retained for the UK sample. Profiles were also interpretable: Profile 1 reflected higher conscientiousness, lower neuroticism, higher NFC, lower PSMU and FoMO, and higher SA; Profile 2 showed the opposite pattern, with lower conscientiousness and NFC, higher neuroticism and lower SA; Profile 3 was characterized by average traits but elevated PSMU and FoMO. Agreement between *k*-means and LPA was stronger in the UK sample (ARI = 0.64). Cross-classification indicated that all three clusters corresponded closely to their respective LPA profiles, with minimal misclassification. Overall, this supports a stable and interpretable three-cluster structure in the UK sample. (See Section G in the Appendix in the OSF link provided in the ‘Data accessibility’ section for full standardized means and cross-classification results).

#### 3.2.2. From cluster to persona creation

To enhance the clarity of the clustering results, each cluster is illustrated through a persona, an archetype that captures the key psychological and behavioural traits of individuals within that group. This method connects statistical analysis with practical relevance, providing an intuitive lens for understanding variations in cybersecurity attitudes and digital behaviour. By portraying data-informed user types, personas make the findings more accessible to diverse stakeholders: designers can develop user-centred solutions, practitioners can customize interventions, and policymakers can design more effective awareness initiatives.

The personas offer a tangible and interpretable representation of the clusters identified in our analysis, translating complex, multi-dimensional patterns into actionable insights. Each persona reflects the core characteristics of its respective cluster, including personality traits (e.g. conscientiousness and neuroticism), NFC, PSMU, FoMO, SA and demographic attributes (age and gender) derived directly from the clustering results. Construct labels such as ‘high’, ‘moderate’ or ‘low’ were determined based on the cluster characteristics, relative to cluster means, to ensure consistency and avoid arbitrary assignments. Age and gender were not assigned arbitrarily but were selected to mirror the distributional patterns observed within each cluster. The inclusion of names, age and gender aims to enhance interpretability and usability; these elements are adaptable and are not intended as fixed or factual representations beyond the data. Persona representations may vary depending on context and methodological choices, emphasizing that their descriptions, including accompanying images, are flexible rather than fixed [74]. Illustrative features such as names, facial representations and clothing style are included solely to support interpretation and usability and are explicitly not meant to depict real individuals.

Personas are not intended to provide exact, one-to-one representations of statistical clusters or results; instead, they function as flexible templates that summarize key patterns for interpretation and practical application. This approach is consistent with established practices in human–computer interaction (HCI), human-centred design and behavioural research. For example, a study by Calde *et al*. defines personas as fictional, detailed archetypes representing groups of similar users [75], while another study by Salminen *et al*. describes personas as representative constructs that facilitate empathy and communication rather than enforcing strict segmentation [76].

Personas serve as a bridge between quantitative analysis and practical application, supporting design decisions [77]. Recent research increasingly frames personas not only as user-centred design tools but also as actionable artefacts that inform decision-making in policy, healthcare and social interventions. For instance, Gonzalez de Heredia *et al.* demonstrated that personas are employed in policy-making and healthcare contexts, aiding institutions such as the UK Government's Open Policy Making toolkit and organizations like TACSI in communicating population needs and designing appropriate interventions [78]. Within policy design frameworks, personas function as artefacts that guide the development, evaluation and communication of policy ideas by providing meaningful archetypes against which solutions can be tested. Creating a persona allows stakeholders to engage with behavioural and psychological profiles holistically, something that is often challenging to achieve through numerical tables or cluster labels alone. Moreover, personas are living artefacts that can be adapted or refined in future research, highlighting the flexibility and broader applicability of our findings across contexts.

## 4. Discussion

This paper introduces a novel, data-driven approach to constructing user personas based on psychological, emotional and cognitive traits. Personas are widely employed in app development and policy design to define target groups and capture their unique characteristics. Developing detailed, evidence-based personas supports user well-being by enabling the design of digital experiences tailored to varied emotional and cognitive needs [79], for example by considering mental health and emotional states to reduce stress and enhance engagement. Previous research illustrates the utility of personas in health and behaviour-focused design. LeRouge *et al.* used personas to model the mental frameworks of ageing populations in China, informing health app development [80], while Holden *et al.* applied personas to understand the needs of older adults with heart failure in user-centred interventions [81]. Similarly, Altuwairiqi *et al.* developed five behavioural archetypes reflecting diverse attachment styles to guide personalized tools for behaviour change, supporting creativity, customization and communication in the design process [82].

Personas are not intended to replace academic analysis or obscure empirical findings; rather, they are a well-established tool in design science and HCI for translating analytical results into interpretable representations that support communication, brainstorming and design decision-making [83,84]. As AI-based design and evaluation tools become increasingly common, personas provide a structured means of operationalizing user differences, supporting testing of system acceptance, anticipated impact and potential interventions, while enhancing explainability and communication in AI-assisted systems [85]. Importantly, personas can also serve as agentive representations, allowing users to be mapped based on observed behaviour or enabling systems to dynamically infer personas and adapt content, interactions or interventions accordingly. In the following sections, we discuss each persona in detail, highlighting their distinctive characteristics and implications for social media awareness and cyber behaviour strategies.

### 4.1. Analysis of user profiles

#### 4.1.1. UK personas

**Figure 3.**
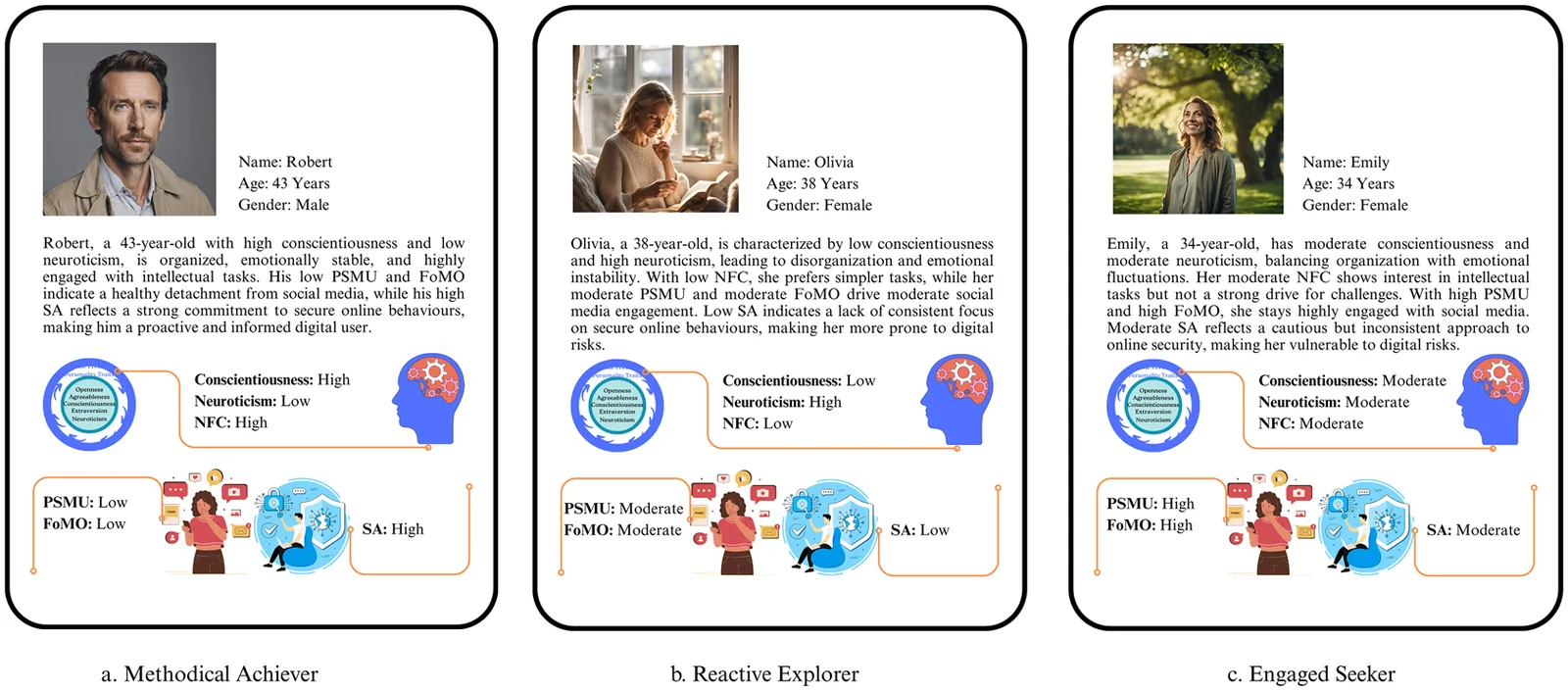
UK personas.

For the UK clusters, three different user archetypes were identified, as shown in figure 3. In UK Cluster 1, termed ‘methodical achiever’, this archetype, represented by a middle-aged man named ‘Robert’, reflects a persona defined by careful planning, emotional stability and a proactive approach to online safety. Aligning with previous research, Robert's high conscientiousness reflects strong organizational skills and a preference for structured, goal-oriented behaviours [52], which is associated with adherence to best practices in managing digital security. Consistent with previous research, his low neuroticism indicates emotional stability, reducing his likelihood of experiencing stress or anxiety [53,86] when confronted with online risks. This emotional resilience allows him to navigate the digital landscape confidently, clearly avoiding impulsive or emotionally driven decisions. Robert's high NFC reveals a strong preference for mentally stimulating tasks and a desire to engage deeply with complex information [42]. This cognitive engagement makes him more likely to critically evaluate online risks and adopt evidence-based solutions to protect his digital privacy and security. His intellectual curiosity aligns with findings from Cacioppo & Petty, who demonstrated that individuals with high NFC are more motivated to process detailed information thoroughly [42]. His low PSMU and low FoMO suggest reduced engagement in passive social media use, which aligns with Mao & Zhang, who found that lower passive browsing may contribute to lower levels of trait FoMO [87]. Robert uses digital tools purposefully rather than compulsively, maintaining control over his online habits. This minimizes his vulnerability to the emotional pressures and impulsive behaviours often linked to excessive social media use. According to Maathuis & Chockalingam, Robert's high SA underscores his commitment to secure and responsible digital behaviour [88]. He takes proactive steps to safeguard his data, such as using strong passwords, enabling multi-factor authentication and staying informed about the latest cybersecurity practices.

In UK Cluster 2, termed ‘reactive explorer’, this archetype, represented by a middle-aged woman named ‘Olivia‘, reflects a persona defined by emotional volatility and impulsive decision-making in the digital landscape. Olivia's low conscientiousness reveals a tendency towards disorganization and difficulty with goal setting, which affects her ability to adopt structured or consistent security behaviours. Research on conscientiousness suggests that it plays an essential role in adherence to systematic approaches for managing risks, particularly in health-related behaviours [89]. Given the parallels between structured risk management in health and digital security, individuals low in conscientiousness may similarly struggle with maintaining consistent cybersecurity practices. Aligning with findings by Thompson [90], her high neuroticism suggests frequent emotional instability, making her more likely to experience stress and anxiety, in situations involving online risks. Her low NFC means she prefers straightforward tasks and avoids cognitively demanding challenges, often relying on heuristics or impulsive responses in her decision-making. This aligns with findings from Cacioppo & Petty's work on NFC, highlighting the reduced motivation to process complex information among individuals with low cognitive engagement [42]. As a result, Olivia is less likely to critically evaluate the risks of online interactions, increasing her susceptibility to phishing scams and other digital threats. Moderate levels of PSMU and FoMO reflect occasional reliance on social media and digital platforms for engagement but suggest that these behaviours do not completely consume Olivia. However, these moderate tendencies, combined with her high neuroticism, may amplify stress related to social comparisons or the fear of being left out, especially during emotionally charged situations. For instance, research by Settles *et al*. demonstrates that individuals with similar traits, high neuroticism and low conscientiousness are prone to negative urgency, leading to impulsive reactions under stress [91]. Olivia's low SA signals limited attention to cybersecurity practices, such as using weak passwords or neglecting updates, which places her at further risk. Her approach to digital security reflects a reactive rather than proactive stance, driven by immediate emotional responses rather than long-term planning.

In UK Cluster 3, termed ‘engaged seeker’, this archetype, represented by a young woman named ‘Emily’, highlights a persona actively engaged with digital platforms but with mixed emotional and cognitive traits that influence her online behaviours. Emily's moderate conscientiousness suggests a balanced approach to organization and planning. While she can be methodical in certain areas, she may occasionally struggle with maintaining consistency in her online security practices. Her moderate neuroticism indicates emotional variability, making her prone to stress or anxiety in uncertain situations, but not to the extent of overwhelming instability. Emily's moderate NFC reflects a willingness to engage in cognitively demanding tasks when necessary, though she may not consistently seek out challenging or thought-provoking activities. This aligns with her ability to analyse and adapt to new digital tools and risks, albeit somewhat cautiously. Aligning with a previous study, Emily's high PSMU and high FoMO reveal a strong attachment to social media and digital interactions [92,93]. She frequently engages with social platforms, driven to stay connected and avoid missing out on social updates. These tendencies can sometimes lead to compulsive behaviours, such as overchecking notifications or spending excessive time online, potentially impacting her emotional well-being [94]. Research by Przybylski *et al.* highlights that high FoMO often correlates with increased social media use, contributing to feelings of dependency and digital fatigue [12]. Her moderate SA suggests an awareness of cybersecurity practices, but with inconsistent application. While Emily may understand the importance of secure online behaviour, her high PSMU and FoMO could detract from her ability to implement these practices consistently. This creates a dynamic where emotional and social pressures might overshadow her rational understanding of online risks. A study found that individuals with higher trust in Internet technology and vendors are more inclined to use social media and online shopping platforms, sometimes at the expense of rigorous security practices [95].

#### 4.1.2. Arab personas

**Figure 4.**
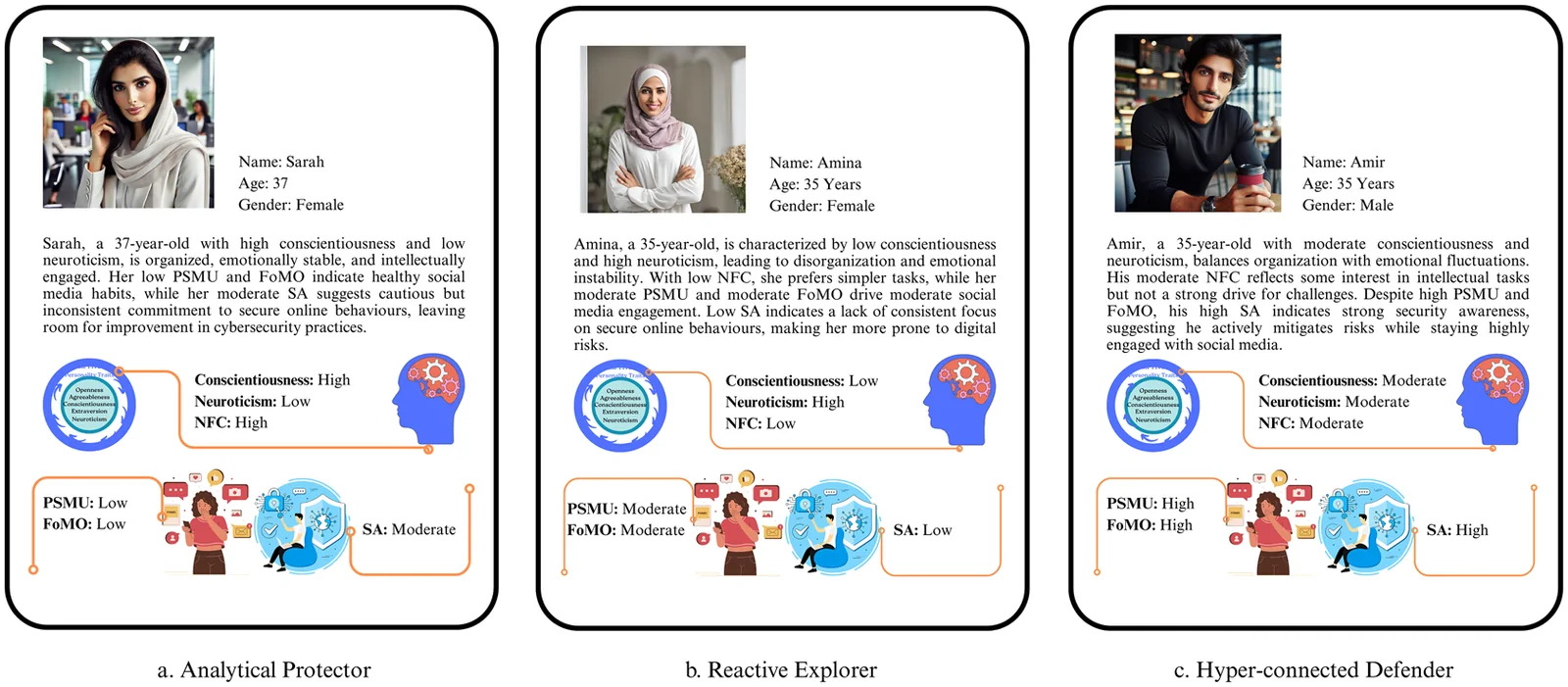
Arab personas. *Note:* The visual layout and presentation of the persona profiles were created with Canva (www.canva.com), while illustrative facial representations were generated with the AI-based tool Fotor (www.fotor.com). These images were used solely for visualization purposes and do not contribute to the analysis. The images do not represent real individuals and were not intended to convey or manipulate demographic characteristics; therefore, they should not be interpreted as reflecting actual participants or introducing demographic bias.

Based on the characteristics of the three clusters in the Arab sample, we can identify distinct personas that reflect their behaviours, motivations and challenges, as shown in figure 4. Cluster 1 is represented by ‘Sarah’, the persona we termed ‘analytical protector’, a 36-year-old female characterized by high conscientiousness, low neuroticism and a strong NFC. This profile aligns with previous research suggesting that highly conscientious individuals exhibit greater organizational skills and a more methodical approach to decision-making [96]. Sarah's low neuroticism and high emotional stability further support findings that individuals with these traits are less likely to react impulsively or experience stress in the face of uncertainty, which might result in more rational processing of risks [97]. Sarah's minimal reliance on social media (low PSMU) and limited susceptibility to FoMO are consistent with research linking lower levels of PSMU and FoMO with high self-control and a preference for more structured, predictable environments [98]. These traits also align with studies indicating that individuals less engaged with social media are less likely to experience heightened vulnerability to online threats or manipulation [99]. However, while Sarah demonstrates a moderate SA, her rational and emotionally stable nature might lead her to underestimate certain cybersecurity risks, as individuals with lower emotional reactivity may be less attuned to perceived threats. The existing literature on cybersecurity behaviour suggests that individuals with high NFC tend to engage in more thoughtful analysis of security information [43], but they may prioritize efficiency over perceived risk. Thus, interventions for Sarah should focus on providing structured, logical guidelines that underscore the practical benefits of cybersecurity practices. Research by Ifinedo [100] has highlighted that individuals like Sarah, who are analytical and methodical, are more likely to adopt security measures when these measures emphasize tangible, logical advantages.

Cluster 2, embodied by ‘Amina’, is the ‘reactive explorer’, a 35-year-old female who mirrors the characteristics seen in the UK cluster who struggles with organization and is prone to stress (low conscientiousness and high neuroticism). With moderate levels of social media dependency and FoMO, Amina's low-security attitude makes her more vulnerable to social engineering and phishing attacks. Effective strategies for Amina should focus on reducing her cognitive load by simplifying security practices and appealing to her emotions to underscore the importance of safety.

Finally, Cluster 3, the ‘hyper-connected defender’ persona, exemplified by ‘Amir’, shares several key psychological traits that inform his cybersecurity behaviours. At 35 years old, Amir displays moderate conscientiousness, neuroticism and NFC, which positions him as a balanced figure in the digital landscape. His moderate conscientiousness allows for some degree of organization but not to the extent of highly structured individuals. However, his moderate neuroticism suggests that he may still experience stress in online interactions, though it does not significantly detract from his cybersecurity practices. Amir's high engagement with social media and intense FoMO align with high levels of PSMU [101]. This profile is similar to that of individuals who rely heavily on social platforms to maintain connections and stay informed [102]. In contrast to other personas, Amir balances this high digital engagement with a high SA. These findings contradict prior research that revealed users with ‘careless’ and ‘carefree’ attitudes, particularly those highly engaged on social media, tend to have lower security concerns and are more likely to explore and exploit various applications without stringent security considerations [103]. Amir might be an exception owing to factors such as higher cybersecurity awareness, education or intrinsic motivation to maintain secure behaviours despite high engagement. His proactive stance towards safeguarding his online presence reflects a more intentional approach to digital security, as he actively seeks to implement protective measures, such as strong passwords and regular software updates. The findings from the current research suggest that individuals like Amir, who exhibit high PSMU and FoMO, are often vulnerable to digital threats [23,104] owing to impulsive behaviours driven by emotional responses and social pressures. However, Amir's high SA counters this tendency by motivating him to take deliberate actions to reduce risks. This aligns with existing research, which indicates that higher SAs are associated with proactive cybersecurity behaviours [105]. Similarly, studies suggest that individuals with high levels of PSMU and FoMO, like Amir, may experience increased susceptibility to online manipulation and phishing scams [104]. However, Amir's elevated security awareness reduces his risk exposure. Studies show how education or targeted interventions can mitigate risks associated with high digital engagement [106].

A key contribution of this study is the practical application of the created personas to develop targeted cybersecurity interventions. Traditional, one-size-fits-all strategies often fail to address different user groups’ diverse needs and perceptions. By using personas that represent distinct segments of the population, this research allows for the design of more personalized and effective interventions tailored to specific emotional, cognitive and behavioural profiles [79]. This approach enhances the effectiveness of cybersecurity strategies by directly addressing the unique risk perceptions and behaviours of each persona. The clustering approach offers context-specific insights, particularly when comparing UK and Arab populations. By accounting for cultural factors, this study enables the development of culturally sensitive interventions, recognizing how these variables intersect to shape cybersecurity behaviours [107]. Additionally, this study moves beyond demographic analysis by considering factors like the NFC, PSMU and FoMO, providing a multi-dimensional perspective on how individuals interact with digital security behaviour. This approach is essential for understanding how individuals respond to security challenges and interventions.

Our clustering analysis challenges prior assumptions regarding the relationship between conscientiousness, neuroticism, PSMU, FoMO and SA. While the existing literature suggests that individuals with high PSMU and FoMO generally exhibit low SA [26,27], our findings reveal a more nuanced structure. Specifically, only one cluster aligned with this expectation, whereas other clusters demonstrated moderate-to-high levels across all three dimensions. This indicates that high PSMU and FoMO do not universally predict weak security behaviours, suggesting the need for more tailored interventions that account for varying psychological profiles. Additionally, the distribution of cluster sizes was relatively comparable, highlighting that the assumed negative association between PSMU, FoMO and SA is not as prevalent as previously thought. Unlike cyber behaviour, which displayed cluster-specific variations, personality traits followed expected patterns, such as low conscientiousness and low NFC, which aligned with high neuroticism or vice versa. However, the way these personality profiles translated into cyber behaviours was not symmetrical; for example, individuals with low conscientiousness, low NFC and high neuroticism did not exhibit cyber behaviours that were the direct inverse of those with high conscientiousness, high NFC and low neuroticism. This underscores the complexity of how psychological traits interact with digital security behaviours, reinforcing the importance of moving beyond one-size-fits-all frameworks in cybersecurity interventions. This aligns with evidence from research on small and medium-sized enterprises, which also shows that generic, one-size-fits-all strategies are ineffective and that tailored approaches, sensitive to context and maturity, are essential [108].

In addition to its novel contributions, this study is subject to several limitations that should be acknowledged. First, the reliance on self-reported data may introduce response bias, despite measures taken to ensure participant anonymity and voluntary participation. Second, although the inclusion of UK and Arab samples provides valuable cross-cultural insight, the findings may not readily generalize to other cultural contexts. We excluded non-binary participants owing to small sample sizes and participants aged 60 and above in the UK sample to ensure comparability with the age distribution of the Arab GCC sample, as we were not able to recruit sufficient participants from this age group in the Arab dataset, rather than this being an intentional exclusion criterion. While these decisions were necessary to maintain analytic consistency and cluster comparability, they may limit the generalizability of the findings. In particular, the exclusion of these groups may have influenced the resulting cluster structure and its interpretation, as patterns present within these populations were not captured in the present analysis. Future research should therefore include more diverse age ranges and gender identities to further test the stability of cluster solutions and enhance the external validity of the findings across broader populations.

While these decisions were necessary to maintain analytic consistency and cluster comparability, they may limit the generalizability of the findings. In particular, the exclusion of these groups may have influenced the resulting cluster structure and its interpretation, as patterns present within these populations were not captured in the present analysis. Future research should therefore include more diverse age ranges and gender identities to further test the stability of cluster solutions and enhance the external validity of the findings across broader populations.

The reliance on quantitative methods may not fully capture the richness and complexity of individual experiences, which could be more deeply explored through qualitative approaches. An additional limitation concerns the current application of the personas. Although they are grounded in empirical data and offer a useful interpretive framework, their practical applicability for designers, therapists or policymakers has not yet been empirically validated. Accordingly, future research should prioritize stakeholder engagement to evaluate how these personas can be translated into practical applications. Such validation is necessary to ensure that the personas are not only theoretically robust but also actionable and relevant in real-world contexts.

## 5. Conclusion

This study developed a clustering approach to examine how personality traits, cognitive styles and cybersecurity-related behaviours intersect across UK and Arab samples. By identifying three distinct personas within each cultural context, we provide a structured and empirically grounded representation of how psychological and behavioural factors jointly shape digital SAs and vulnerabilities. A key contribution of this work is the demonstration that cybersecurity behaviour cannot be adequately explained by isolated traits or a linear relationship. Our results show that combinations of conscientiousness, NFC, FoMO, PSMU and SAs interact in ways that produce distinct and sometimes unexpected behavioural profiles. Notably, patterns typically reported in the literature, such as the association between higher FoMO or PSMU and weaker SAs, do not consistently hold across all clusters. This suggests that digital vulnerability is not fixed at the level of single traits but emerges from broader psychological configurations and contextual interdependencies.

Our findings extend existing research by integrating personality, cognitive motivation and digital behaviour within a single person-centred analytical framework. In doing so, we challenge approaches that explain cybersecurity behaviour by looking at single variables as separate risk factors. Our results suggest that similar levels of a trait can be linked to different cybersecurity outcomes depending on how it appears alongside other psychological characteristics. This shifts the focus from individual predictors to combinations of traits working together.

The novelty of this paper lies in the personas derived in this study, which offer more than descriptive groupings. They represent actionable profiles that can inform the design of tailored cybersecurity interventions. We argue that translating complex empirical patterns into interpretable user archetypes provides a more realistic foundation for intervention design than one-size-fits-all approaches, which often overlook variation in motivation and behaviour.

Nevertheless, these findings should be interpreted in light of the study's limitations. The study does not establish causal relationships, and the cross-sectional, self-reported nature of the data limits the strength of causal inference. While the inclusion of UK and Arab samples strengthens cross-cultural insight, the results should not be assumed to generalize beyond these contexts. Rather than offering a definitive explanatory model, this work should be seen as a step towards a more nuanced, person-centred understanding of cybersecurity behaviour grounded in psychological profiles.

## Data Availability

The dataset associated with this work is uploaded alongside the appendix and other supplementary material for this article at [109].
